# Influence of Support Surface Stability on Changes in Quadriceps Femoris Muscle Thickness During Progressive Squat Exercise in Patients with Stroke: A Randomized Controlled Trial

**DOI:** 10.3390/medicina62091749

**Published:** 2026-09-11

**Authors:** Hui Ju Nam, Ga-Yeon Kim

**Affiliations:** 1 Chungdam Hospital, 46, Samseong-ro 147-gil, Gangnam-gu, Seoul 06063, Republic of Korea; nhj1396@naver.com; 2 Department of Public Health, Graduate School, Dankook University, 119 Dandae-ro, Dongnan-gu, Cheonan-si 31116, Republic of Korea

**Keywords:** quadriceps femoris, stroke, unstable support surface, progressive squat exercise, rehabilitative ultrasound imaging

## Abstract

*Background and Objectives*: Stroke, a neurological disorder caused by damage to the central nervous system, is commonly accompanied by impairments in motor and sensory function and muscle strength. We compared the effects of progressive squat exercises performed under varying support-surface stability conditions on quadriceps femoris muscle thickness in patients 6 months to less than 1 year after stroke, using rehabilitative ultrasound imaging. *Materials and Methods*: This randomized, open-label, parallel-group controlled trial assessed 36 patients with stroke for eligibility. Four individuals were excluded before randomization; the remaining 32 participants were randomly assigned in a 1:1 ratio to either the unstable-support-surface squat exercise group (*n* = 16) or the stable-support-surface squat exercise group (*n* = 16). Both groups performed progressive squat exercises five times per week for 4 weeks. Quadriceps femoris muscle thickness was measured before and after the intervention using rehabilitative ultrasound imaging. *Results*: Between-group comparisons of changes from baseline showed significantly greater increases in rectus femoris and vastus medialis muscle thickness in the unstable support-surface squat exercise group than in the stable support-surface squat exercise group (*p* < 0.05). No significant between-group differences were observed in vastus intermedius or vastus lateralis muscle thickness (*p* > 0.05). *Conclusions*: Progressive squat exercise performed on an unstable support surface resulted in greater increases in rectus femoris and vastus medialis muscle thickness than the same exercise performed on a stable support surface. However, these findings are limited to muscle thickness and should not be interpreted as evidence of improved muscle strength or functional performance.

## 1. Introduction

Stroke is a neurological disorder caused by damage to the central nervous system and is commonly accompanied by impairments in motor and sensory function, as well as muscle strength. Following onset, many patients experience persistent functional disabilities, such as hemiparesis [1,2].

These neurological impairments can lead to lower-limb muscle weakness and atrophy, which may subsequently limit the performance of basic functional activities, including standing and mobility [3,4].

Among the rehabilitation exercises designed to improve lower-limb muscle strength, the progressive squat is a closed-kinetic-chain exercise that requires coordinated movement of multiple joints and muscles. This induces functional muscle recruitment patterns under weight-bearing conditions [5,6]. Additionally, squat exercises involve relatively simple movements and have high clinical applicability; therefore, they are widely used in rehabilitation settings.

Recently, alongside modifying exercise intensity or the number of repetitions, approaches that enhance neuromuscular stimulation by altering the environmental conditions under which the exercise is performed have received increasing attention. Among these approaches, modifying support-surface stability can increase postural control demands and facilitate sensory and motor integration [7,8]. Exercise performed on an unstable support surface increases proprioceptive sensory input and alters muscle recruitment strategies, thereby eliciting greater neuromuscular responses than exercises performed on a stable support surface [9].

Muscle activation patterns during progressive squat exercises may also differ according to support surface stability. Performing squat exercises on an unstable support surface tends to increase lower-limb muscle activation [8,9]. However, research has focused primarily on electromyographic analysis, and relatively few studies have directly examined changes in skeletal muscle thickness following exercise performed under different support-surface conditions. Ultrasound-derived quadriceps muscle thickness has been associated with quadriceps muscle strength in individuals with chronic stroke [10] and with physical function in patients with subacute stroke [11]. Rehabilitative ultrasound imaging (RUSI) is noninvasive, allows repeated measurements, and provides a quantitative assessment of muscle thickness [12,13]. In particular, the quadriceps femoris is a major muscle group involved in lower-limb functional performance, and the reliability and validity of ultrasound-based measurements of quadriceps femoris muscle thickness have been reported [14,15].

However, there is insufficient evidence regarding changes in quadriceps femoris muscle thickness following progressive squat exercises performed under different support-surface stability conditions in patients 6 months to less than 1 year after stroke. The present analysis focused specifically on quadriceps femoris muscle thickness because RUSI enables the noninvasive and quantitative assessment of changes in the individual components of the quadriceps femoris. Functional outcomes were not included within the scope of the present analysis; therefore, the findings were interpreted specifically in relation to changes in muscle thickness and not as evidence of improvements in functional performance. Accordingly, this study aimed to compare changes in affected-side quadriceps femoris muscle thickness following a 4-week progressive squat exercise program performed on unstable and stable support surfaces in these patients. We hypothesized that progressive squat exercise performed on an unstable support surface would result in greater increases in affected-side quadriceps femoris muscle thickness than the same exercise performed on a stable support surface.

## 2. Materials and Methods

### 2.1. Study Design and Trial Registration

This study was a single-center, open-label, two-arm, parallel-group randomized controlled trial with a 1:1 allocation ratio. The trial was retrospectively registered with the Clinical Research Information Service (CRIS), Republic of Korea (KCT0012481) on 19 August 2026. The delayed registration resulted from an unintentional administrative oversight related to the investigators’ misunderstanding of the prospective public registration requirement for this investigator-initiated, non-pharmacological rehabilitation exercise trial. However, the study protocol had been prospectively prepared and submitted for institutional ethical review before participant enrollment. The first participant was enrolled on 26 June 2023, and the study was completed on 28 August 2023.

### 2.2. Participants

Thirty-six patients with stroke admitted to the C Rehabilitation Hospital in Seoul, Republic of Korea, were assessed for eligibility. Four individuals were excluded before randomization because of COVID-19 infection (*n* = 1), peritonitis (*n* = 1), guardian refusal to participate (*n* = 1), and stent insertion surgery (*n* = 1). The remaining 32 participants were randomly assigned to either the unstable support-surface squat exercise group (*n* = 16) or the stable support-surface squat exercise group (*n* = 16). All randomized participants completed the 4-week intervention and were included in the final analysis. No participants were lost to follow-up or discontinued the intervention after randomization. The inclusion criteria were 6 months to less than 1 year after stroke onset, a Functional Ambulation Category (FAC) score of ≥1, a Berg Balance Scale (BBS) score of ≥41, a Mini-Mental State Examination—Korea (MMSE-K) score of ≥24, no significant visual, auditory, or vestibular impairment, and the ability to understand the study procedures and provide informed consent. The exclusion criteria were orthostatic hypotension, receipt of robot-assisted therapy in addition to conventional therapy, scheduled discharge during the study period, or a history of surgery due to bone fracture. Although eligibility was restricted to patients who were 6 months to less than 1 year after stroke onset, participant-level data on stroke type, lesion location, and exact time since onset were not extracted or included in the dataset prepared for the present muscle-thickness analysis. This study was approved by the Institutional Review Board of Sahmyook University (IRB No. SYU 2023-05-013-002) on 20 June 2023, and written informed consent was obtained from all participants before participation in the study.

### 2.3. Randomization and Blinding

The principal investigator generated the random allocation sequence using a computer-generated random number table and was also responsible for participant enrollment and group assignment in a 1:1 ratio. No independent allocator or allocation-concealment mechanism was used; therefore, group assignments were not concealed from the enrolling investigator. Because of the nature of the intervention, participants and the treating investigator were not blinded to group allocation; therefore, the study was open-label.

### 2.4. Sample Size Calculation

The sample size was calculated a priori using G*Power software (version 3.1.9.2). Based on Lee et al. [6], published before data collection, the effect size was set to 0.70. With a significance level of 0.05 and statistical power of 0.80, a minimum of 32 participants, with 16 participants per group, was required. Allowing for an anticipated attrition rate of approximately 10%, 36 patients were assessed for eligibility.

### 2.5. Intervention

Both groups performed progressive squat exercises for 30 min per session, five sessions per week, for 4 weeks. The exercise program consisted of five sets of 10 repetitions during weeks 1–2 and six sets of 10 repetitions during weeks 3–4. A 60 s rest period was provided between sets. The unstable support-surface group performed the exercises on a balance pad, whereas the stable support-surface group performed the same exercises on a flat surface. For fall-risk management, the principal investigator stood on the affected side within arm’s reach without maintaining physical contact and was prepared to provide assistance if required for safety. A treatment table was positioned immediately behind the participant, and its height was adjusted to correspond to 90° of knee flexion so that the participant could sit if necessary. Falls and other intervention-related adverse events were monitored throughout the 4-week intervention.

In addition to the study intervention, both groups received neurodevelopmental treatment (NDT) as part of conventional physical therapy according to a physician’s prescription. NDT consisted of therapeutic approaches based on neurodevelopmental and motor-learning principles and was provided for 30 min per session, twice daily, 10 sessions per week, for 4 weeks. Both groups also received routine occupational therapy for 30 min per session, twice daily, 10 sessions per week, for 4 weeks. The frequency and duration of both NDT and occupational therapy were identical between the two groups.

### 2.6. Measurement of Quadriceps Femoris Muscle Thickness

Quadriceps femoris muscle thickness on the affected side was measured at baseline and after the 4-week intervention using a RUSI system (MySono U5; Samsung Medison, Seoul, Republic of Korea) equipped with an 8 MHz linear-array transducer. The gain was fixed at 40 and maintained consistently for all participants and assessment time points. Measurements were performed using a standardized protocol adapted from a previously published quadriceps ultrasound measurement procedure [16].

Participants were positioned supine on a treatment table with both knees fully extended and the lower-extremity muscles relaxed. At each assessment, the measurement sites were identified using the same anatomical landmarks and proportional distances and were marked on the skin with an indelible pen. Measurements were performed in the following order: rectus femoris, vastus intermedius, vastus lateralis, and vastus medialis.

The rectus femoris and vastus intermedius were measured at 50% of the distance between the anterior superior iliac spine and the superior border of the patella. The vastus lateralis was measured at the same longitudinal level, at a point located laterally by 10% of the thigh circumference from the rectus femoris and vastus intermedius measurement site. The vastus medialis was measured at 20% of the distance from the superior border of the patella toward the anterior superior iliac spine and at a point located medially by 12.5% of the thigh circumference.

At each marked measurement site, the linear-array transducer was positioned in the transverse plane, with the long axis of the transducer oriented perpendicular to the longitudinal axis of the thigh. The transducer was maintained perpendicular to the skin and muscle surface during image acquisition. A sufficient amount of water-soluble ultrasound gel was applied between the transducer and the skin. To minimize compression of the underlying soft tissue, the transducer was applied with minimal pressure sufficient only to maintain acoustic contact, and excessive compression or visible deformation of the muscle was avoided during image acquisition. The central vertical line of each ultrasound image was used consistently for muscle-thickness measurement. Rectus femoris, vastus lateralis, and vastus medialis thicknesses were measured at the central vertical caliper line as the perpendicular distance between the inner borders of the superficial and deep fascial interfaces. Vastus intermedius thickness was measured at the same central vertical line as the perpendicular distance between the deep fascial boundary of the rectus femoris and the superficial border of the femoral cortex.

All baseline and post-intervention ultrasound measurements were performed by the same principal investigator, a physical therapist with 6 years of clinical experience, including extensive experience using ultrasound imaging for muscle measurements, who had completed ultrasound training. Before data collection, the examiner performed repeated practice sessions using the same ultrasound equipment and measurement protocol to standardize probe placement, image acquisition, and muscle-thickness measurement procedures. Each muscle was measured three times at each assessment, and the mean of the three measurements was used as the final value for statistical analysis. Study-specific intra-rater reliability was not formally assessed. Because all measurements were performed by a single examiner, inter-rater reliability was not evaluated.

### 2.7. Data Analysis

Data are presented as means and standard deviations. The outcome variables were the muscle thicknesses of the rectus femoris, vastus intermedius, vastus lateralis, and vastus medialis. For each participant and each muscle, the change score was calculated as the post-intervention value minus the baseline value. Within-group changes from baseline to post-intervention were analyzed using paired-samples *t*-tests. Between-group comparisons were performed using independent-samples *t*-tests applied to the change scores, rather than to the raw post-intervention values. Welch’s *t*-test was used when the assumption of equal variances was not satisfied. For the primary between-group comparisons of change scores, mean differences with 95% confidence intervals were calculated using the same independent-samples *t*-test framework, with the Welch–Satterthwaite approximation applied when the assumption of equal variances was not satisfied. Hedges’ g, calculated from the pooled standard deviation of the change scores with a small-sample correction, was used to quantify the standardized magnitude of the between-group differences. Positive mean differences and Hedges’ g values indicated greater increases in the unstable support-surface group. To control the family-wise error rate across the four primary between-group muscle-thickness comparisons, the *p* values from the change-score analyses were adjusted using the Holm–Bonferroni procedure. As a post hoc sensitivity analysis, analysis of covariance (ANCOVA) was performed separately for each muscle, with post-intervention muscle thickness as the dependent variable, group as the fixed factor, and the corresponding baseline muscle thickness as the covariate. The homogeneity-of-regression-slopes assumption was examined using the group-by-baseline interaction. The Holm–Bonferroni procedure was also applied to the four group-effect *p* values obtained from the ANCOVA models. All statistical tests were two-sided, and statistical significance was set at *p* < 0.05. Statistical analyses were performed using SPSS version 23.0.

## 3. Results

### 3.1. General Characteristics of the Participants

**Figure 1:**
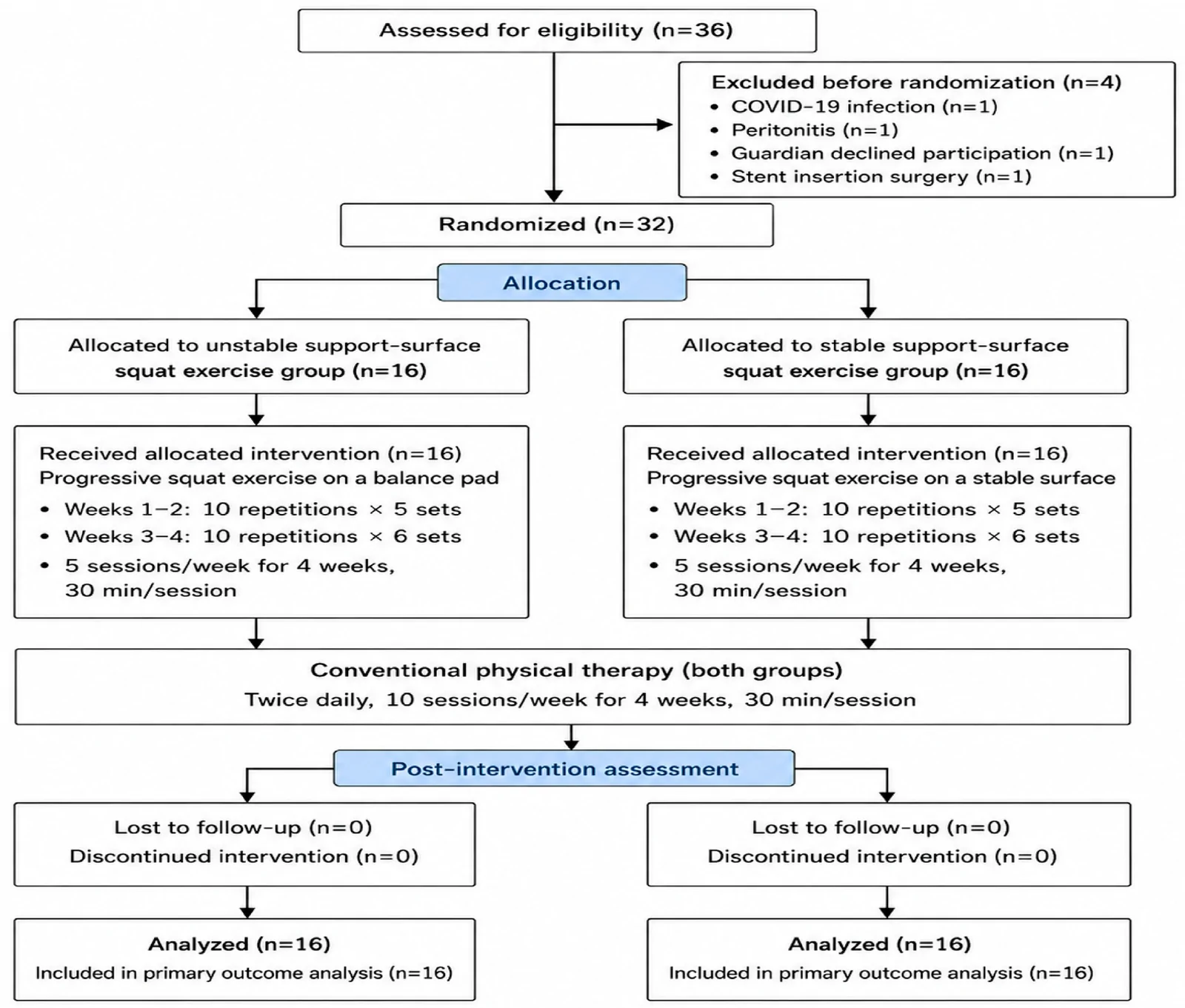
CONSORT flow diagram of participant enrollment, allocation, follow-up, and analysis.

Thirty-six patients were assessed for eligibility. Four individuals were excluded before randomization, and the remaining 32 participants were randomly assigned to the unstable support-surface group (*n* = 16) or the stable support-surface group (*n* = 16). All randomized participants completed the intervention and were included in the final analysis. The participant flow is shown in Figure 1. No falls or other intervention-related adverse events were observed in either group during the study period.

The general characteristics of the participants are presented in Table 1. The unstable support-surface squat exercise group comprised 11 males and 5 females, with a mean age of 65.31 years, whereas the stable support-surface squat exercise group comprised 8 males and 8 females, with a mean age of 62.81 years. No statistically significant between-group differences were found in the measured baseline characteristics; however, numerical differences were present in sex distribution and body weight.

### 3.2. Changes in Quadriceps Femoris Muscle Thickness

**Figure 2:**
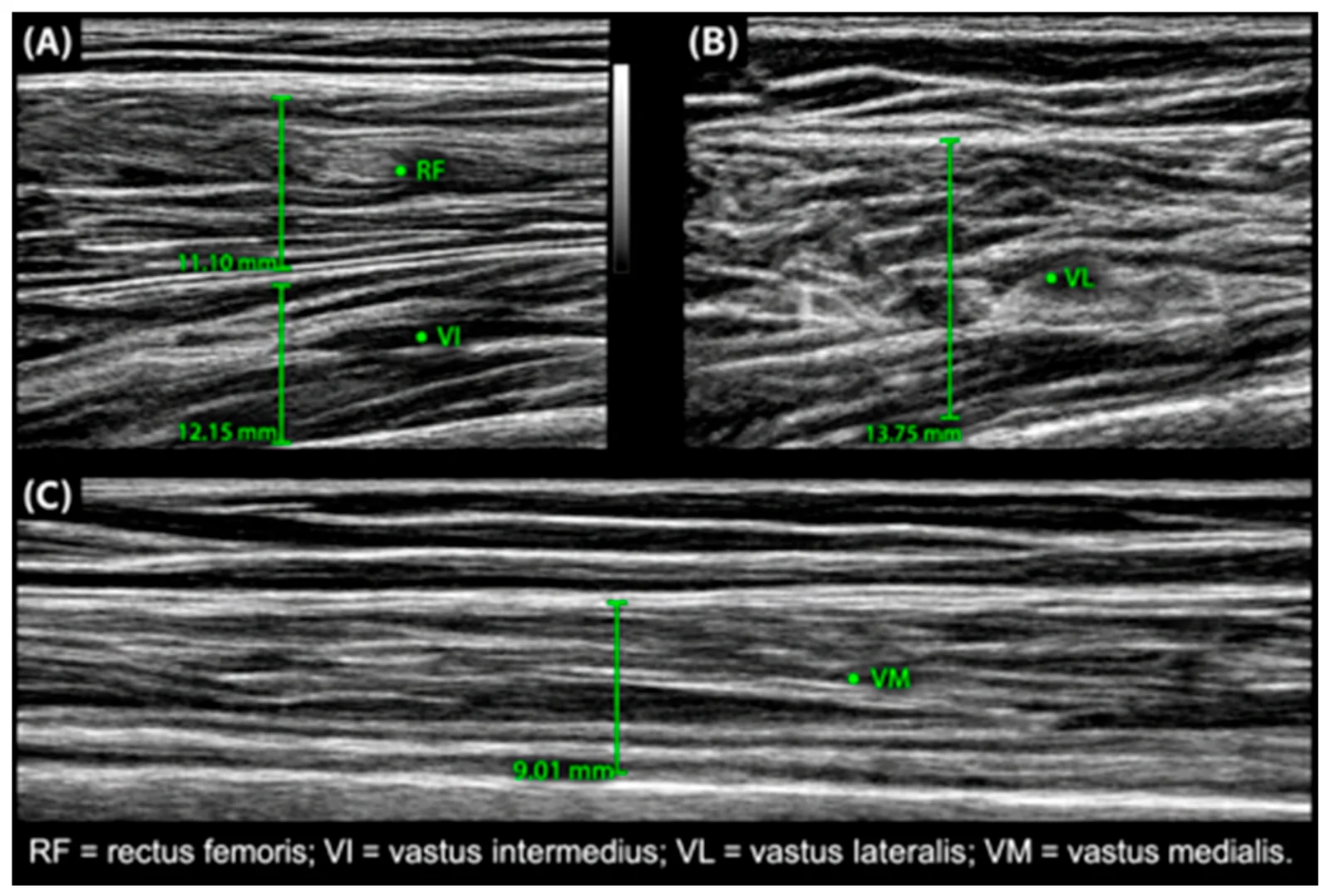
Representative RUSI images of quadriceps femoris muscle-thickness measurements. (**A**) Rectus femoris and vastus intermedius; (**B**) vastus lateralis; and (**C**) vastus medialis. Each panel represents a distinct ultrasound acquisition obtained at the corresponding anatomical measurement site. Muscle thickness was measured at the central vertical caliper line.

Quadriceps femoris muscle thickness was measured at baseline and after the 4-week intervention using RUSI. Representative ultrasound images used for the muscle-thickness measurements are shown in Figure 2.

Between-group comparisons of the change scores for the four quadriceps femoris muscles, including mean differences, 95% confidence intervals, effect sizes, and Holm-adjusted *p*-values, are summarized in Table 2.

### 3.3. Changes in Rectus Femoris Muscle Thickness

**Figure 3:**
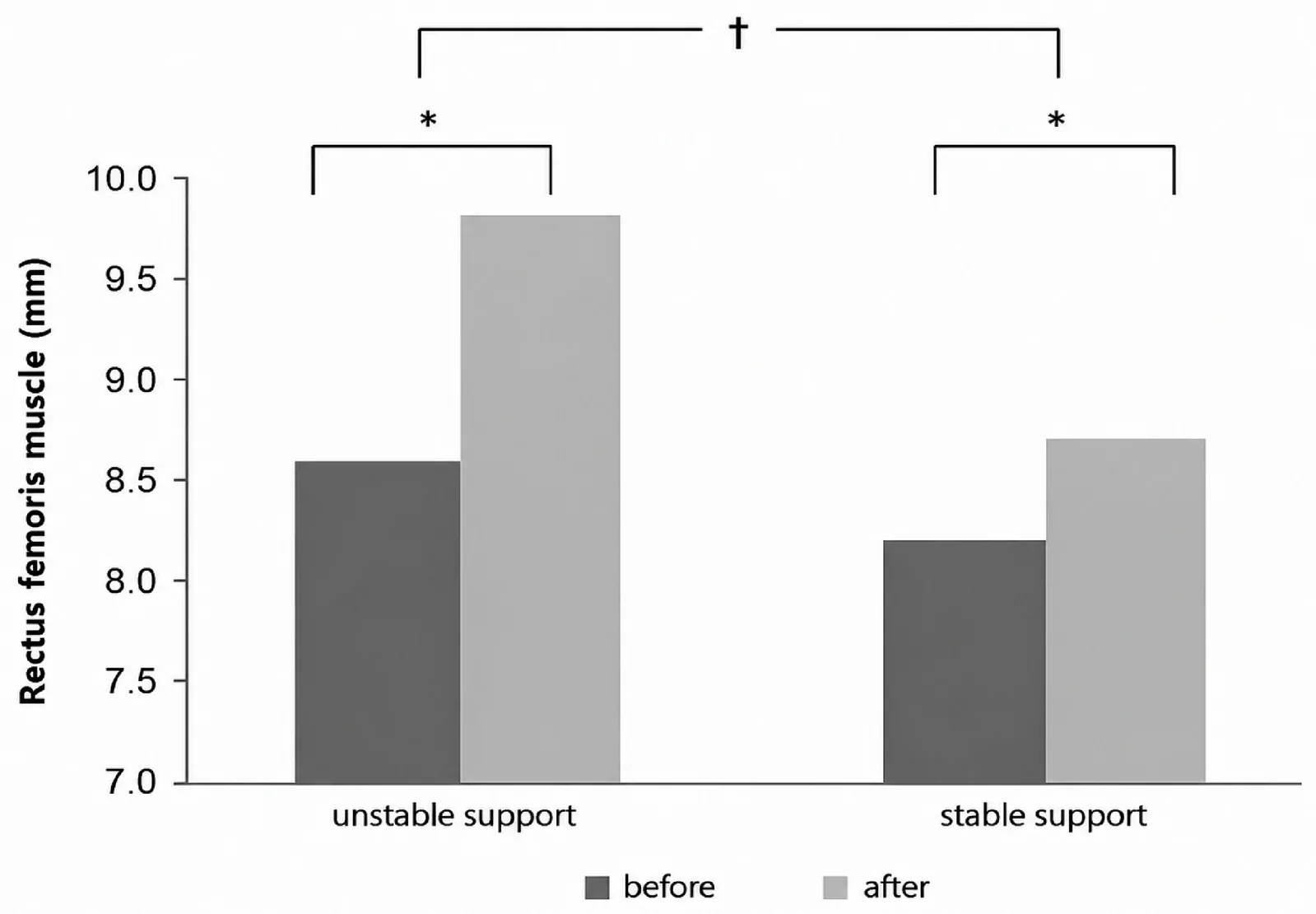
Changes in rectus femoris muscle thickness from baseline to post-intervention in the unstable and stable support-surface groups. * *p* < 0.05 for within-group pre–post comparisons; † Holm-adjusted *p* < 0.05, when present, for between-group comparisons of change scores.

Pre- and post-intervention changes in rectus femoris muscle thickness in the unstable and stable support surface squat exercise groups are shown in Figure 3.

In the unstable support-surface squat exercise group, rectus femoris muscle thickness increased significantly from 8.58 mm before intervention to 9.85 mm after intervention (*p* < 0.001). In the stable support surface squat exercise group, rectus femoris muscle thickness also increased significantly from 8.22 mm before intervention to 8.71 mm after intervention (*p* < 0.001).

The mean change in rectus femoris muscle thickness was 1.27 ± 0.79 mm in the unstable support-surface group and 0.49 ± 0.33 mm in the stable support-surface group. The between-group difference in change was 0.78 mm (95% CI, 0.34 to 1.23; Hedges’ g = 1.27), with a significantly greater increase in the unstable support-surface group. This difference remained significant after Holm–Bonferroni correction (unadjusted *p* = 0.002; Holm-adjusted *p* = 0.006).

### 3.4. Changes in Vastus Intermedius Muscle Thickness

**Figure 4:**
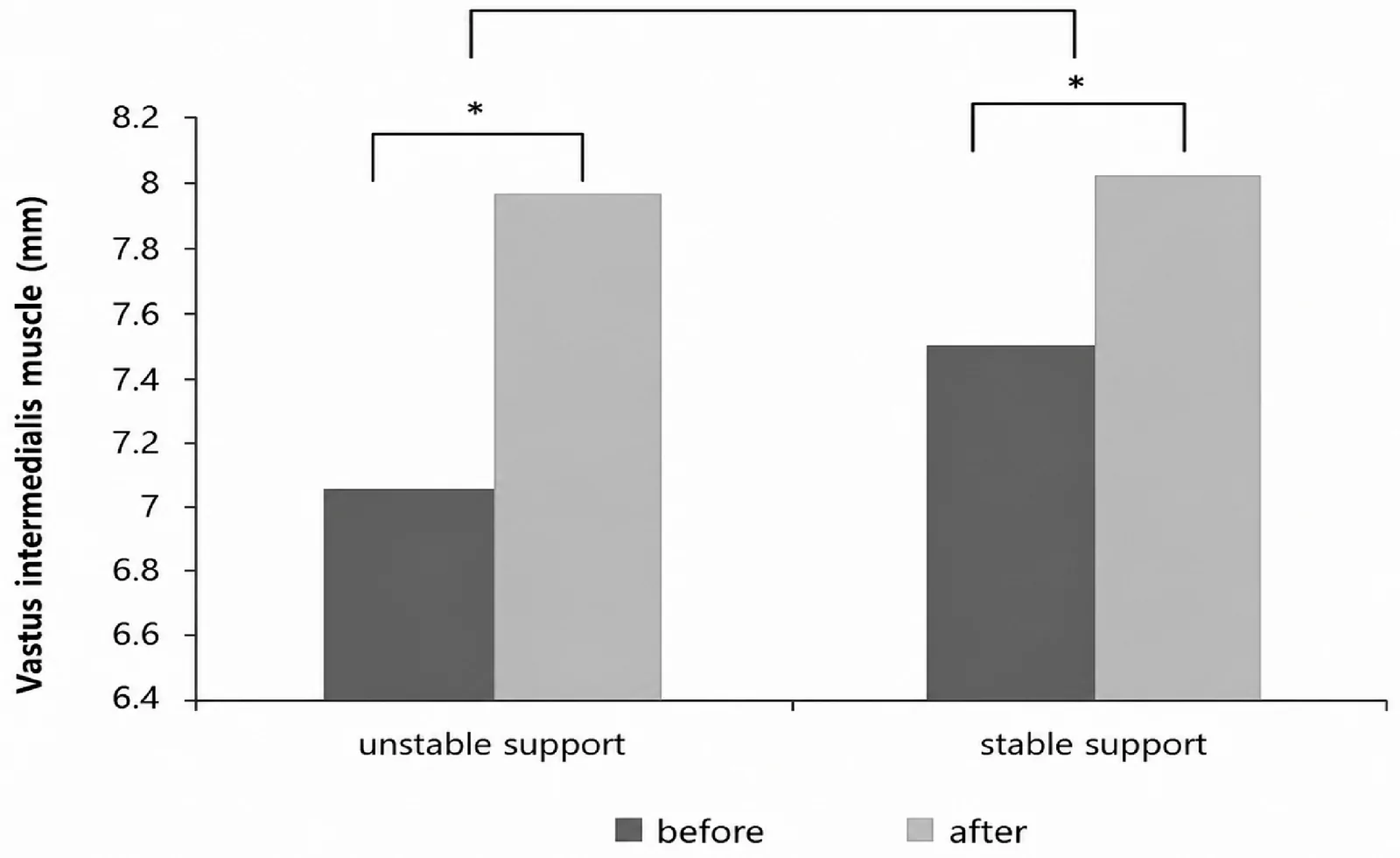
Changes in vastus intermedius muscle thickness from baseline to post-intervention in the unstable and stable support-surface groups. * *p* < 0.05 for within-group pre–post comparisons.

Pre- and post-intervention changes in vastus intermedius muscle thickness in the unstable and stable support surface squat exercise groups are shown in Figure 4.

In the unstable support-surface squat exercise group, vastus intermedius muscle thickness increased significantly from 7.06 mm at baseline to 7.97 mm after the intervention (*p* = 0.031). In the stable support-surface squat exercise group, vastus intermedius muscle thickness also increased significantly from 7.51 mm at baseline to 8.03 mm after the intervention (*p* = 0.005).

The mean change was 0.91 ± 1.53 mm in the unstable support-surface group and 0.52 ± 0.63 mm in the stable support-surface group. The between-group difference in change was 0.39 mm (95% CI, −0.45 to 1.23; Hedges’ g = 0.32). No significant between-group difference was observed in the change scores after Holm–Bonferroni correction (unadjusted *p* = 0.353; Holm-adjusted *p* = 0.353).

### 3.5. Changes in Vastus Lateralis Muscle Thickness

**Figure 5:**
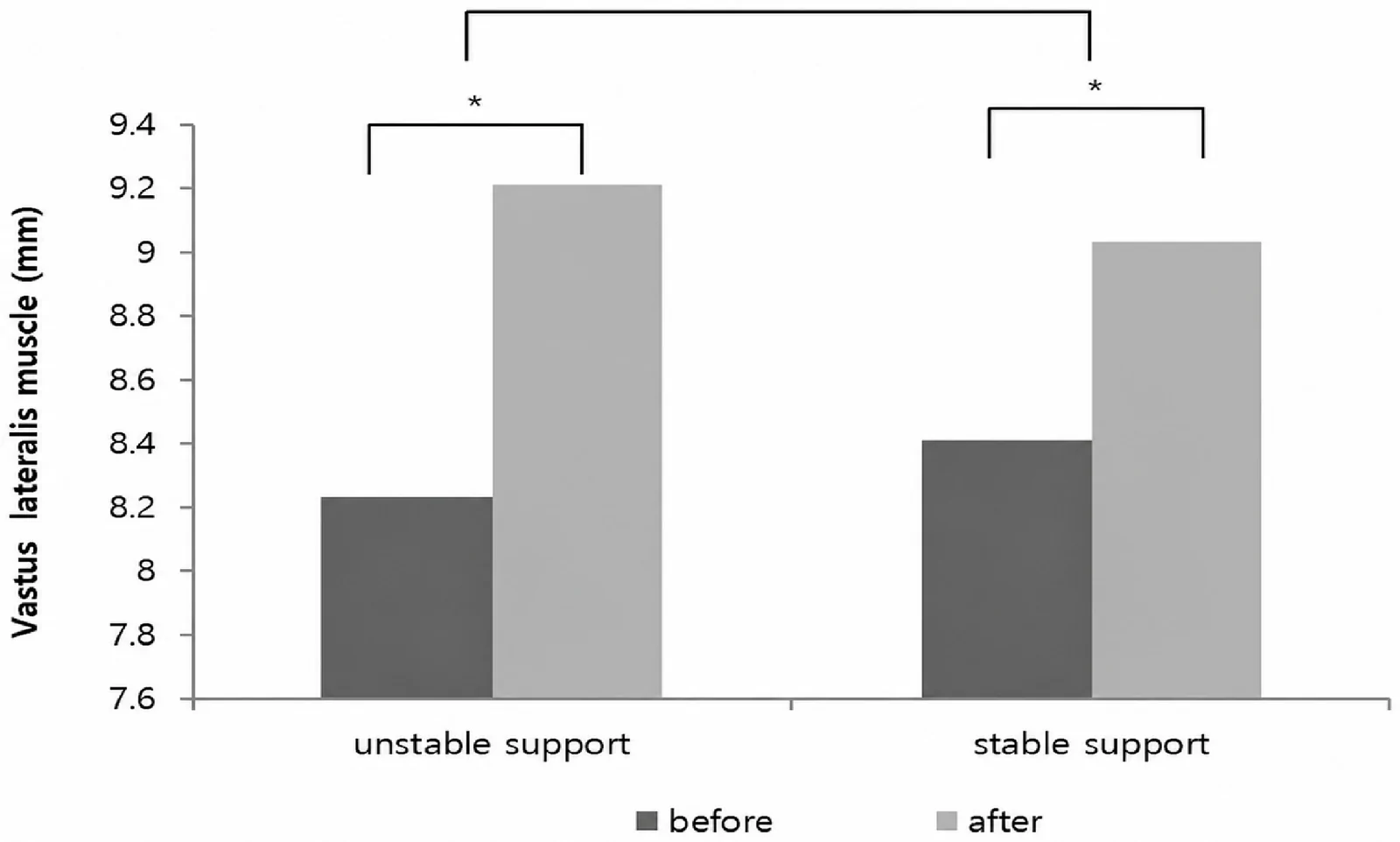
Changes in vastus lateralis muscle thickness from baseline to post-intervention in the unstable and stable support-surface groups. * *p* < 0.05 for within-group pre–post comparisons.

Pre- and post-intervention changes in vastus lateralis muscle thickness in the unstable and stable support surface squat exercise groups are shown in Figure 5.

In the unstable support-surface squat exercise group, the vastus lateralis muscle thickness increased significantly from 8.23 mm before intervention to 9.21 mm after intervention (*p* < 0.001). In the stable support surface squat exercise group, vastus lateralis muscle thickness significantly increased from 8.41 mm before the intervention to 9.03 mm after the intervention (*p* < 0.001).

The mean change in vastus lateralis muscle thickness was 0.98 ± 0.66 mm in the unstable support-surface group and 0.62 ± 0.50 mm in the stable support-surface group. The between-group difference in change was 0.36 mm (95% CI, −0.06 to 0.78; Hedges’ g = 0.60). No significant between-group difference was observed after Holm–Bonferroni correction (unadjusted *p* = 0.090; Holm-adjusted *p* = 0.181).

### 3.6. Changes in Vastus Medialis Muscle Thickness

**Figure 6:**
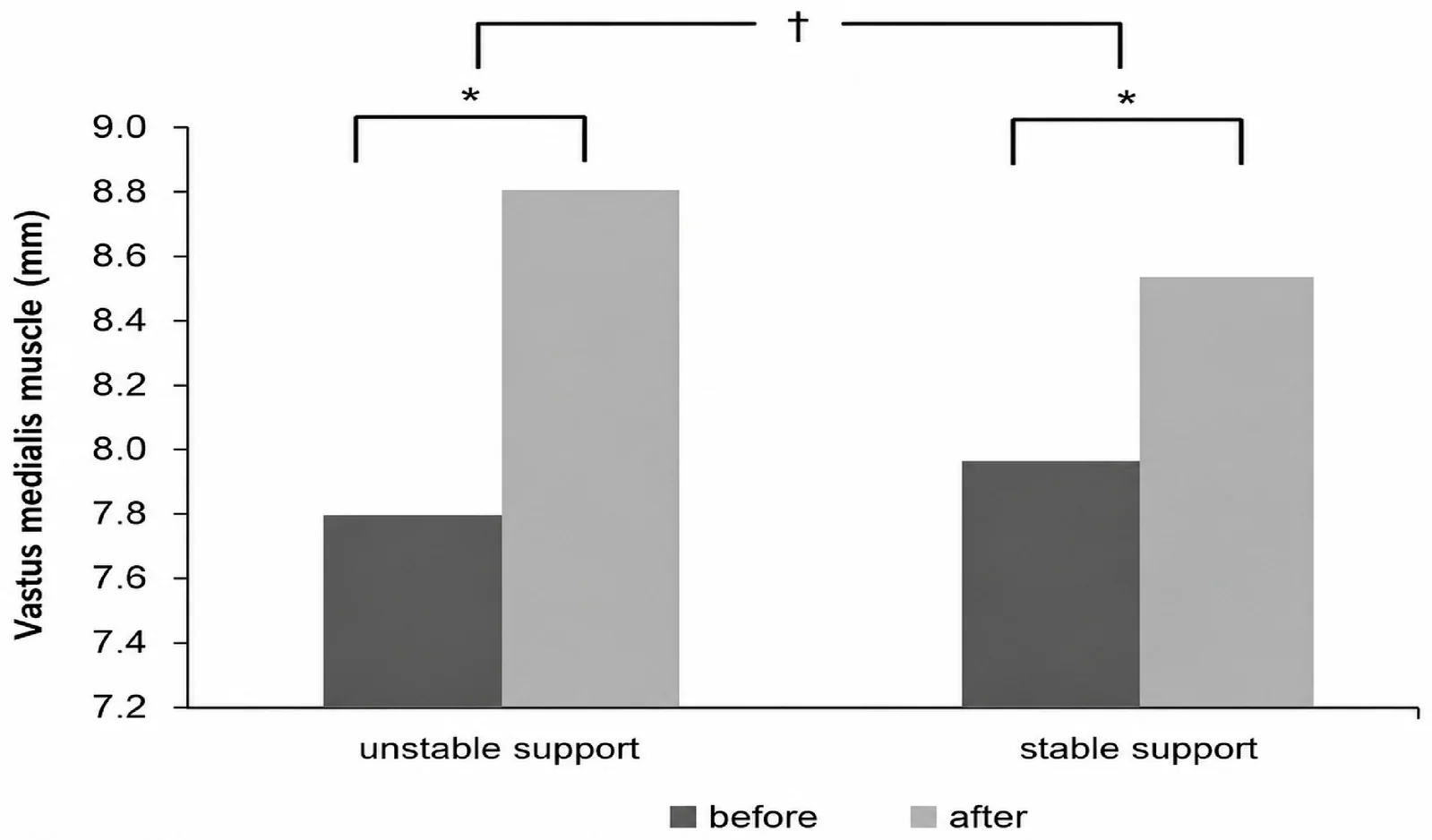
Changes in vastus medialis muscle thickness from baseline to post-intervention in the unstable and stable support-surface groups. * *p* < 0.05 for within-group pre–post comparisons; † Holm-adjusted *p* < 0.05, when present, for between-group comparisons of change scores.

The pre- and post-intervention changes in the vastus medialis muscle thickness in the unstable and stable support surface squat exercise groups are shown in Figure 6.

In the unstable support-surface squat exercise group, vastus medialis muscle thickness increased significantly from 7.78 mm at baseline to 8.81 mm after the intervention (*p* < 0.001). In the stable support-surface squat exercise group, vastus medialis muscle thickness also increased significantly from 7.95 mm at baseline to 8.53 mm after the intervention (*p* < 0.001).

The mean change in vastus medialis muscle thickness was 1.03 ± 0.60 mm in the unstable support-surface group and 0.58 ± 0.31 mm in the stable support-surface group. The between-group difference in change was 0.44 mm (95% CI, 0.09 to 0.79; Hedges’ g = 0.90), with a significantly greater increase in the unstable support-surface group. This difference remained significant after Holm–Bonferroni correction (unadjusted *p* = 0.015; Holm-adjusted *p* = 0.046).

### 3.7. Sensitivity Analysis

In the baseline-adjusted ANCOVA models, no significant group-by-baseline interactions were observed for any of the four muscles (all *p* > 0.05). After Holm–Bonferroni correction, the adjusted between-group differences in post-intervention muscle thickness were significant for the rectus femoris (adjusted mean difference = 0.78 mm; 95% CI, 0.33 to 1.23; Holm-adjusted *p* = 0.005) and vastus medialis (adjusted mean difference = 0.45 mm; 95% CI, 0.10 to 0.80; Holm-adjusted *p* = 0.042). No significant adjusted between-group differences were observed for the vastus intermedius (adjusted mean difference = 0.41 mm; 95% CI, −0.46 to 1.27; Holm-adjusted *p* = 0.346) or vastus lateralis (adjusted mean difference = 0.35 mm; 95% CI, −0.07 to 0.77; Holm-adjusted *p* = 0.205). These findings were consistent with the primary change-score analysis.

## 4. Discussion

This study showed that quadriceps femoris muscle thickness increased in both groups after the 4-week intervention. After Holm–Bonferroni correction, the unstable support-surface group showed significantly greater increases in rectus femoris and vastus medialis muscle thickness than the stable support-surface group, whereas no significant between-group differences were observed for vastus intermedius or vastus lateralis. Thus, the study hypothesis was partially supported. The between-group differences in change were approximately 0.78 mm for the rectus femoris and 0.44 mm for the vastus medialis. These findings were consistent with the baseline-adjusted ANCOVA; however, their clinical significance remains uncertain because study-specific minimal detectable change values were not established. Because muscle strength and functional performance were not assessed, the observed changes in muscle thickness should not be interpreted as evidence of clinically meaningful functional improvement.

Stuart et al. quantified tibiofemoral joint forces and muscle activity during closed-kinetic-chain exercises, including squat exercises [5]. Lee et al. compared quadriceps femoris and hamstring muscle activity during various squat exercises [6]. Previous studies have primarily examined muscle activation during squat exercises, whereas the present study used RUSI to identify changes in quadriceps femoris muscle thickness following the 4-week exercise intervention.

Rectus femoris and vastus medialis muscle thicknesses increased significantly more in the unstable support-surface group than in the stable support-surface group. Previous studies have shown that unstable support surfaces can increase neuromuscular demands during squat exercises [8,9]. However, muscle activation and mechanical loading were not directly measured in the present study. Therefore, the mechanisms underlying the greater changes in rectus femoris and vastus medialis muscle thickness cannot be determined from the present findings.

In contrast, no significant between-group differences were observed in the changes in vastus intermedius or vastus lateralis muscle thickness. These findings indicate that the responses were not uniform across the four components of the quadriceps femoris. Differences in the architecture and functional roles of the quadriceps femoris muscles may have contributed to these muscle-specific responses [17]. However, because electromyography or other direct neuromuscular measures were not collected, this mechanistic explanation remains speculative and should be interpreted cautiously.

RUSI provides a noninvasive method for quantifying muscle thickness. Chi-Fishman et al. demonstrated that ultrasound imaging could distinguish between normal and weak muscle [12], and Wallwork et al. reported intra- and inter-rater reliability of RUSI for muscle-thickness measurements [13]. Santos and Armada-da-Silva reported the reproducibility of ultrasound-derived muscle-thickness measurements across the quadriceps femoris muscles [14]. Worsley et al. demonstrated the validity of ultrasound measurements of distal vastus medialis thickness compared with magnetic resonance imaging [15]. In the present study, RUSI was used to quantify changes in rectus femoris, vastus intermedius, vastus lateralis, and vastus medialis thickness following the intervention.

This study has several limitations. First, only quadriceps femoris muscle thickness was analyzed, and other muscle architectural characteristics were not evaluated. Second, muscle strength and functional performance were not included as outcomes in the present analysis. Therefore, the observed increases in muscle thickness should not be interpreted as direct evidence of improvements in muscle strength or functional performance. Third, the principal investigator administered the interventions and performed all ultrasound measurements and was not blinded to group allocation. Fourth, participant-level data on stroke type, lesion location, and exact time since onset were not included in the dataset used for the present muscle-thickness analysis. Therefore, potential between-group differences in these stroke characteristics and their influence on muscle-thickness changes could not be evaluated. Fifth, no non-exercise control group was included because the study was designed to directly compare progressive squat exercises performed under two different support-surface conditions. Although eligibility was restricted to patients who were 6 months to less than 1 year after stroke onset, the within-group increases observed in both groups may have been influenced by time-related recovery, concurrent NDT, or routine occupational therapy. The frequency and duration of NDT and occupational therapy were identical between the groups; therefore, these co-interventions were unlikely to explain the between-group differences. However, their potential contribution to the within-group increases cannot be separated from the effects of the squat exercise. Therefore, the present findings demonstrate the relative differences between the two support-surface conditions but do not establish the effect of squat exercise compared with no exercise. Sixth, the relatively small sample size, single-center design, and short 4-week intervention period may limit the generalizability of the findings and do not allow conclusions regarding the persistence of the observed muscle-thickness changes. Seventh, allocation concealment was not implemented, and the principal investigator was responsible for participant enrollment, group assignment, intervention delivery, and ultrasound outcome assessment. Therefore, selection and assessor-related bias cannot be excluded. Finally, although all ultrasound measurements were performed by the same examiner and the mean of three repeated measurements was used, study-specific intra-rater reliability was not quantified, and inter-rater reliability was not evaluated because a single examiner performed all assessments.

## 5. Conclusions

This study found that progressive squat exercise increased quadriceps femoris muscle thickness under both stable and unstable support-surface conditions in patients 6 months to less than 1 year after stroke. The increases in rectus femoris and vastus medialis muscle thickness were significantly greater under the unstable support-surface condition than under the stable support-surface condition. These findings are limited to changes in muscle thickness and should not be interpreted as evidence of improved muscle strength or functional performance.

## Figures and Tables

**Table 1:** General characteristics of study participants (*n* = 32).

	Squat Exercise Group on an Unstable Support Surface (*n* = 16)	Squat Exercise Group on a Stable Support Surface (*n* = 16)	Test Statistic (*p*-Value)
Sex (Male/Female)	11/5	8/8	1.065 (0.29)
Affected side (Rt/Lt)	8/8	8/8	0.000 (1.00)
Age	65.31 ± 11.56	62.81 ± 8.48	0.697 (0.49)
Height (cm)	168.31 ± 8.45	165.75 ± 10.72	0.751 (0.45)
Weight (kg)	61.31 ± 12.09	68.19 ± 13.35	1.527 (0.13)

Data are presented as mean ± standard deviation for continuous variables and as the number of participants for categorical variables. Values in the final column are presented as the test statistic followed by the *p*-value in parentheses.

**Table 2:** Between-group comparisons of changes in quadriceps femoris muscle thickness.

Muscle	Unstable Support-Surface Group, Change (mm)	Stable Support-Surface Group, Change (mm)	Mean Difference (95% CI), mm	Hedges’ g	Unadjusted *p*-Value	Holm-Adjusted *p*-Value
Rectus femoris	1.27 ± 0.79	0.49 ± 0.33	0.78 (0.34 to 1.23)	1.27	0.002	0.006
Vastus intermedius	0.91 ± 1.53	0.52 ± 0.63	0.39 (−0.45 to 1.23)	0.32	0.353	0.353
Vastus lateralis	0.98 ± 0.66	0.62 ± 0.50	0.36 (−0.06 to 0.78)	0.60	0.090	0.181
Vastus medialis	1.03 ± 0.60	0.58 ± 0.31	0.44 (0.09 to 0.79)	0.90	0.015	0.046

Data are presented as mean ± standard deviation unless otherwise indicated. Change scores were calculated as post-intervention minus baseline values. Mean differences were calculated as the change in the unstable support-surface group minus the change in the stable support-surface group. Positive values indicate greater increases in the unstable support-surface group. Hedges’ g was calculated from the between-group difference in change scores using the pooled standard deviation with a small-sample correction. Welch’s *t*-test was used when the assumption of equal variances was not satisfied. *p*-values for the four between-group comparisons were adjusted using the Holm–Bonferroni procedure. CI, confidence interval.

## Data Availability

The data used in this study are available from the corresponding author upon reasonable request. The data are not publicly available due to privacy concerns and ethical restrictions.

## Abbreviations

The following abbreviations are used in this manuscript:

RUSI	rehabilitative ultrasound imaging
